# Coffee and caffeine intake and risk of urinary incontinence: a meta-analysis of observational studies

**DOI:** 10.1186/s12894-016-0178-y

**Published:** 2016-10-06

**Authors:** Shenyou Sun, Dongbin Liu, Ziyao Jiao

**Affiliations:** 1 Department of General surgery, Linyi People’s Hospital, Shandong, 276000 People’s Republic of China; 2 Department of Anesthesiology, Linyi People’s Hospital, Shandong, 276000 People’s Republic of China

**Keywords:** Coffee, Caffeine, Urinary incontinence, Risk, Meta-analysis

## Abstract

**Background:**

Previous results from studies on the relationship between coffee/caffeine consumption and risk of urinary incontinence (UI) are inconclusive. We aim to assess this association using a meta-analysis of observational studies.

**Methods:**

Pertinent studies were identified by searching electronic database (Embase, PubMed and Web of Science) and carefully reviewing the reference lists of pertinent articles until July 2015. Random-effects models were used to derive the summary ORs and corresponding 95 % CIs.

**Results:**

Seven studies (one case-control, two cohort and four cross-sectional) were included in our meta-analysis. The summary ORs for any versus non-consumption were 0.75 (95 % CI 0.54–1.04) for coffee and 1.29 (95 % CI 0.94–1.76) for caffeine consumption. Compared with individuals who never drink coffee, the pooled OR of UI was 0.99 (95 % CI 0.83–1.18) for regular coffee/caffeine drinkers. Coffee/caffeine consumption was not associated with moderate to severe UI (OR 1.18, 95 % CI 0.88–1.58). In stratified analyses by gender, no significant association was found between UI risk and coffee/caffeine consumption in both men (OR 0.99, 95 % CI 0.42–2.32) and women (OR 0.92, 95 % CI 0.80–1.06). By subtype, the pooled ORs were 1.01 (95 % CI 0.86–1.19) for stress UI, 0.99 (95 % CI 0.84–1.16) for urge UI and 0.93 (95 % CI 0.79–1.10) for mixed UI.

**Conclusions:**

This meta-analysis found no evidence for an association between coffee/caffeine consumption and the risk of UI.

## Background

Urinary incontinence (UI) is a common condition with significant impact on overall health and quality of life. It has been estimated that UI prevalence ranged from 5 to 21 % among community dwelling United States men [1–4]. However, UI prevalence estimates differ considerably due to the definition adopted and ranges between 10 % and 40 % among community-dwelling women [5–8]. Although UI is only a symptom of several conditions, ascertaining risk factors would be helpful for identifying high-risk persons and avoidable environmental causes. As for initial UI treatment, lifestyle changes such as fluid modification are strongly recommended.

Coffee and caffeine (coffee/caffeine) are one of the most common beverages worldwide, especially among western countries; thus, investigating its association with various diseases has important public health implications. The relationships between coffee/caffeine and risk of UI have been reported in many studies. However, present epidemiological evidence is inconsistent considering the relationships between coffee/caffeine consumption and the risk of stress, urge and mixed UI. Bortolotti et al. observed no association between coffee and risk of UI in 2000 [9]. Since then, several other studies have been published with inconclusive results [10–12]. For instance, Tettamanti reported that women who often drank coffee had a lower risk of any UI compared to women who did not drink coffee [13]. However, Davis noticed that caffeine consumption was associated with moderate to severe UI in United States men [12].

In order to define the possible associations between coffee/caffeine intake and the risk of UI, we performed a meta-analysis of relevant cohort, case-control and cross-sectional studies.

## Methods

### Search strategy

In performing this meta-analysis, we abided by the Meta-Analysis of Observational Studies in Epidemiology (MOOSE) [14] and preferred reporting items for systematic reviews and meta-analyses (PRISMA) [15] guidelines. Three electronic databases (Medline, Embase and Web of Science) until July 2015 were used for systematic literature search, and search terms included coffee, caffeine, drink, beverage, risk and urinary incontinence. We did not set language or other restrictions in the literature search. As this manuscript is a meta-analysis of available studies, it does not involve ethics and require written informed consent from participants.

### Inclusion criteria

The present meta-analysis only included studies which met the following inclusion criteria: (1) the exposure of interest was coffee or caffeine intake; (2) the outcome of interest was UI; (3) the study design was observational; (4) the study reported adjusted risk estimates with corresponding 95 % CIs for the relationship between coffee/caffeine consumption and risk of UI.

### Data extraction

According to the guidelines for meta-analysis [14], two reviewers independently carried out eligibility evaluation and data extraction. We collected detailed information including year of publication, the name of first author, study design, age and gender of participants, number of cases, exposure, sample size and multivariate adjusted ORs and 95 % CIs for each category of coffee/caffeine intake.

### Statistical analysis

It has been stated that when the outcome was rare, relative risks and ORs could provide similar estimates of risk [16]. In this present meta-analysis, ORs were adopted as a common measure of the association between coffee or caffeine intake and UI risk. In all included studies, the highest level of coffee or caffeine intake was defined as ‘regularly drink coffee’, and the lowest level of coffee or caffeine intake was defined as ‘never drink coffee’. Notably, we only adopted the adjusted OR for this meta-analysis. We derived summary OR estimates with 95 % CIs using the method of DerSimonian and Laird.

To assess heterogeneity among studies, we used the Cochran Q and I^2^ statistics. Subgroup analyses stratified by gender, extent and type of UI were also carried out to explore potential sources of heterogeneity. We evaluated publication bias using a funnel plot and the test proposed by the Begg’s adjusted rank correlation test and by the Egger’s regression test [17, 18]. We carried out statistical analyses using STATA, version 11.0 (STATA, College Station, TX, USA). A *p* value of less than 0.05 was considered statistically significant.

## Results

### Identification of studies

**Fig. 1:**
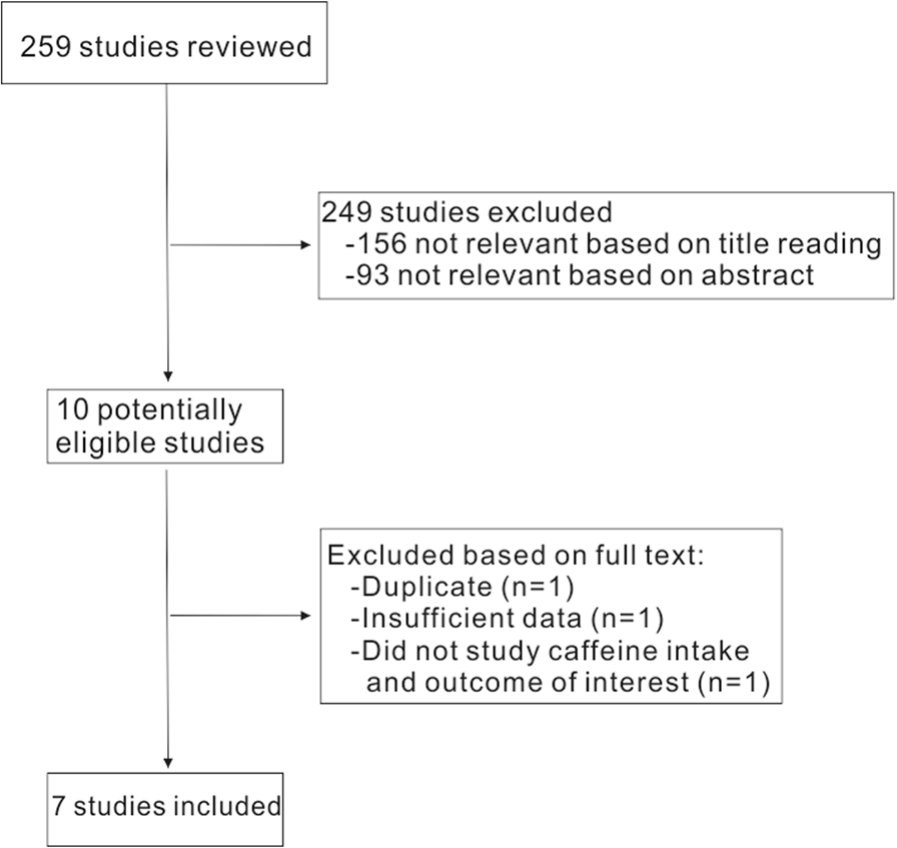
Flowchart of selection of studies for inclusion in the meta-analysis on coffee/caffeine consumption and UI risk

The workflow of the study review is summarized in Fig. 1. A total of 259 studies were retrieved from the initial literature search (61 from the Medline, 167 from the EMBASE, and 31 from the Web of Science). After excluding 249 studies based on title and abstract reading, we reviewed the full texts of the remaining 10 potentially pertinent articles. Finally, seven studies [9–13, 19, 20] which stated the relationship between coffee/caffeine intake and risk UI were included in our meta-analysis. The characteristics of the included studies are shown in Table 1. Among the seven included studies, three reported the data of coffee consumption and four reported caffeine consumption.

**Table 1:** Main characteristics of included studies

First author, year	Country	Study design	Age	Gender	Number of cases	Number of participants	Exposure	Adjustments
Bortolotti, 2000	Italy	Cross-sectional	≥50 (M) ≥40 (F)	Both	408	2721 (M) / 2767 (F)	Coffee	Age
Hannestad, 2003	Norway	Cross-sectional	≥20	Female	6876	27,936	Coffee	Age, BMI and smoking
Jura, 2011	USA	Cohort	37 to 79	Female	15,683	65,176	Caffeine	Age, cohort, parity, BMI, cigarette smoking, race, diabetes, total fluid intake and physical activity
Tettamanti, 2011	Sweden	Cohort	19 to 47	Female	/	14,094	Coffee	Age, parity, BMI, smoking and educational level
Hirayama, 2012	Japan	Case-control	40 to 75	Both	131	683 (M)/298 (F)	caffeine	Age, BMI, smoking status, alcohol drinking, physical activity level, total fluid intake and presence of co-morbidity
Gleason, 2013	USA	Cross-sectional	≥20	Female	1767	4309	Caffeine	Age, race/ethnicity, poverty income ratio, BMI, self-rated health status, major depression, chronic diseases, alcohol use, water intake, total dietary moisture intake and reproductive factors in women including vaginal deliveries
Davis, 2013	USA	Cross-sectional	≥20	Male	511	3960	Caffeine	Age, race/ethnicity, education, BMI, vigorous activity, poverty-to income ratio, chronic disease, health status, depression, alcohol intake, water intake and total moisture intake

### Coffee/caffeine consumption and UI risk

**Fig. 2:**
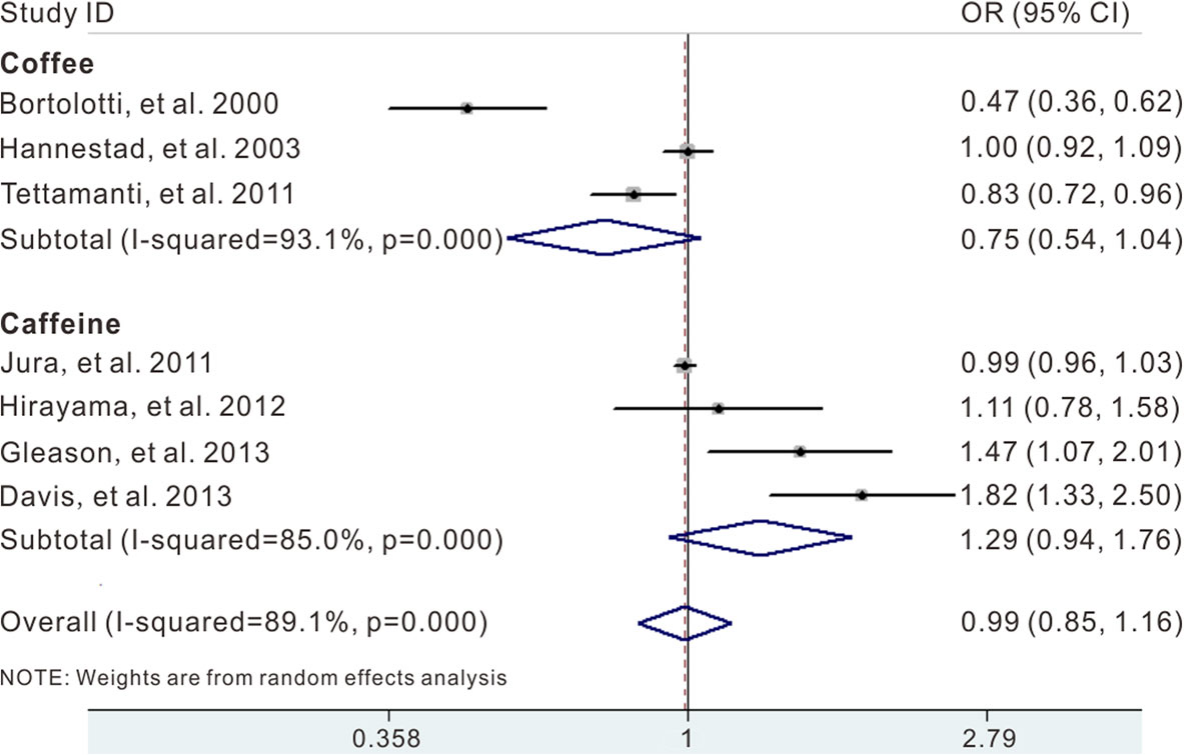
Pooled OR of UI for any versus non-consumption of coffee/caffeine

**Fig. 3:**
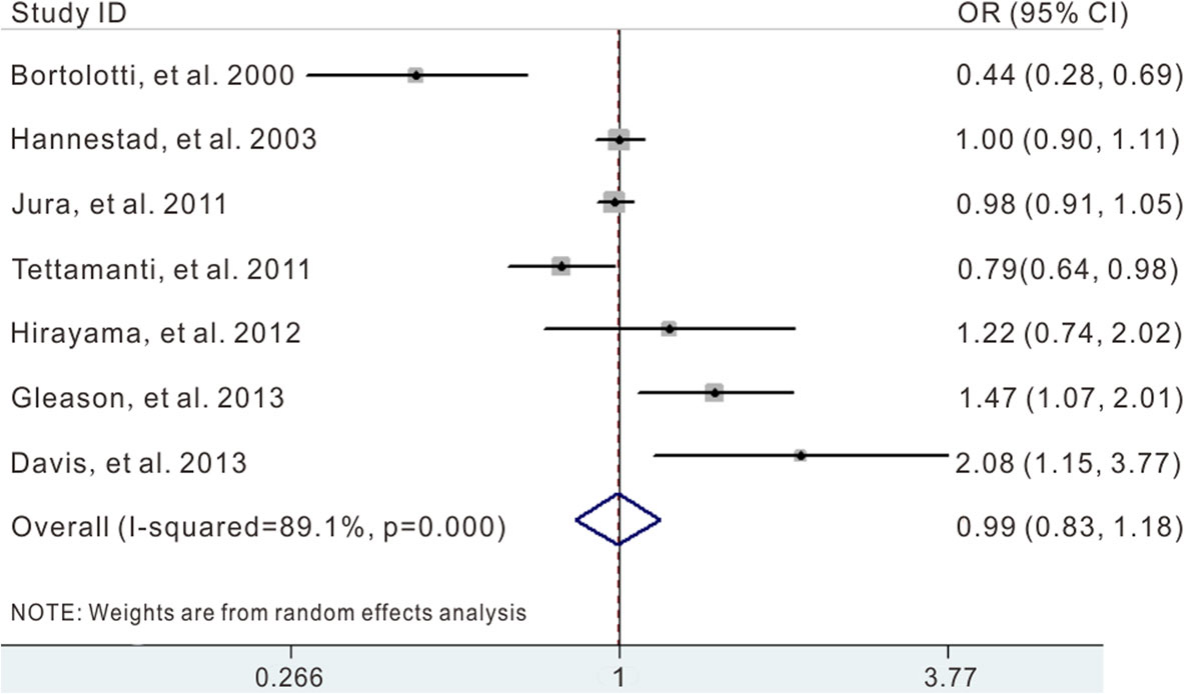
Pooled OR of UI for regular versus non-consumption of coffee/caffeine

The results combining the ORs for the risk of UI associated with coffee/caffeine consumption was summarized in Fig. 2. The summary OR for any versus non-consumption were 0.75 (95 % CI 0.54–1.04) for coffee and 1.29 (95 % CI 0.94–1.76) for caffeine consumption. When combining coffee and caffeine, the summary OR was 0.99 (95 % CI 0.85–1.16) with statistically significant heterogeneity among studies (*I* ^2^ = 89.1 %, *p* = 0.000). Additionally, compared with individuals who never drink coffee, the pooled OR of UI was 0.99 (95 % CI 0.83–1.18) for regular coffee/caffeine drinkers (Fig. 3).

### Coffee /caffeine consumption and incidence of moderate/severe UI

**Fig. 4:**
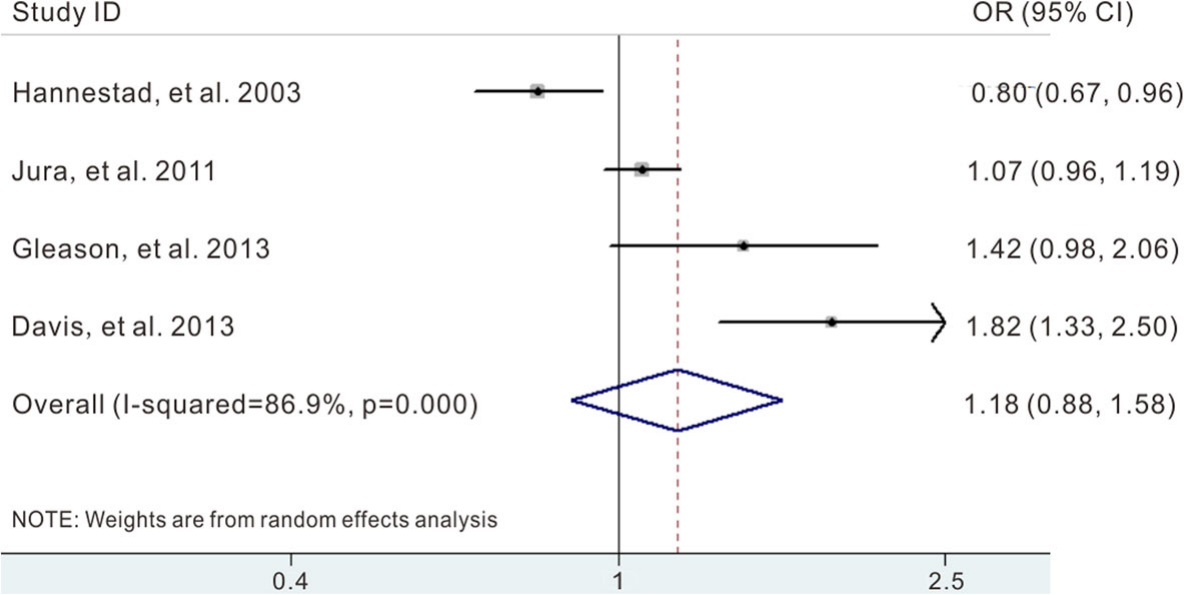
Pooled OR of moderate/severe UI for coffee/caffeine consumption

Three studies provided results on risk of moderate/severe UI [10, 12, 20], and one study reported the risk of frequent UI among women with daily caffeine intakes [11]. In this subgroup meta-analysis, frequent UI was also regarded as moderate/severe UI. The summary OR was 1.18 (95 % CI 0.88 to 1.58) with statistically significant heterogeneity among studies (*I* ^2^ = 86.9 %, *p* = 0.000) (Fig. 4).

### Coffee /caffeine consumption and incidence of UI by sex

**Fig. 5:**
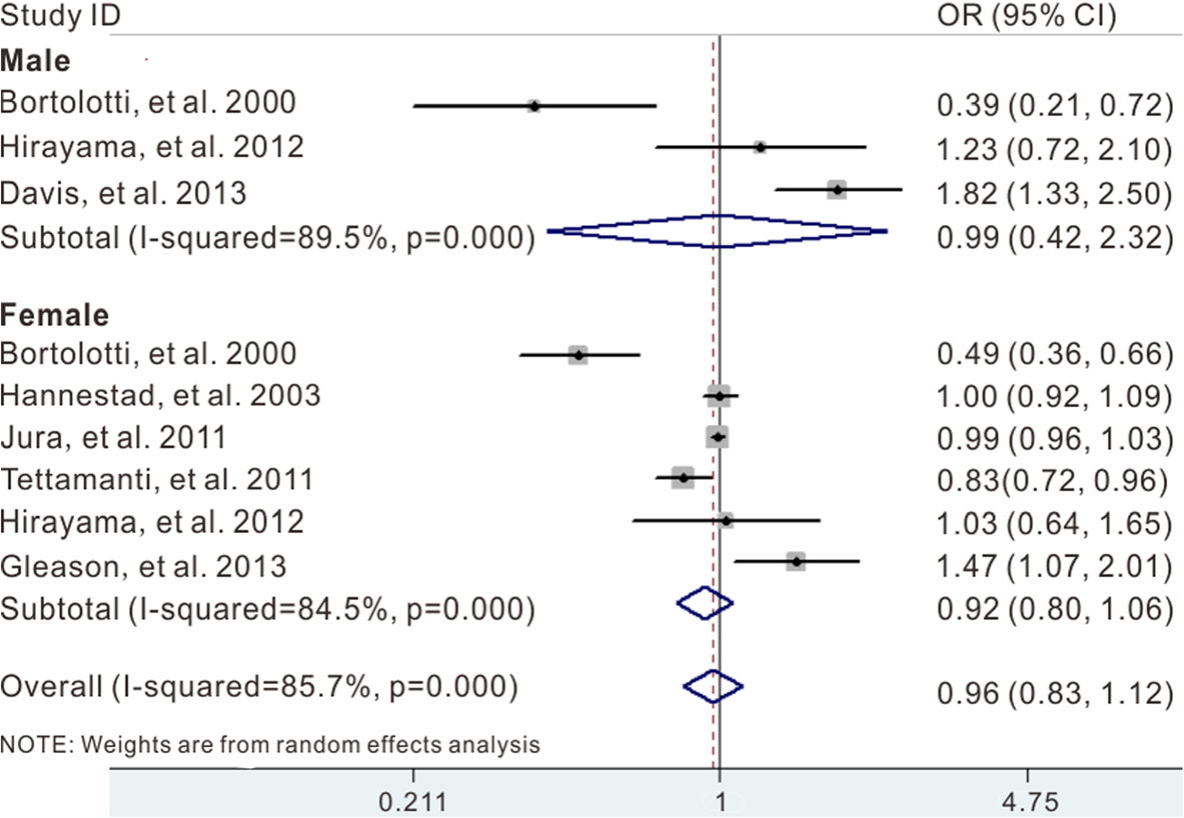
Forest plots of UI risk by gender associated with coffee/caffeine consumption

Two articles reported data on risk of UI specific for gender [9, 19]; one article consisted entirely of men [12] and four articles consisted entirely of women [10, 11, 13, 20]. In stratified analyses by gender, we did not observe any association between coffee/caffeine intake and risk of UI in both men (summary ORs, 0.99; 95 % CI, 0.42–2.32) and women (summary ORs, 0.92; 95 % CI, 0.80–1.06) (Fig. 5).

### Coffee /caffeine consumption and risk of UI by subtype

**Fig. 6:**
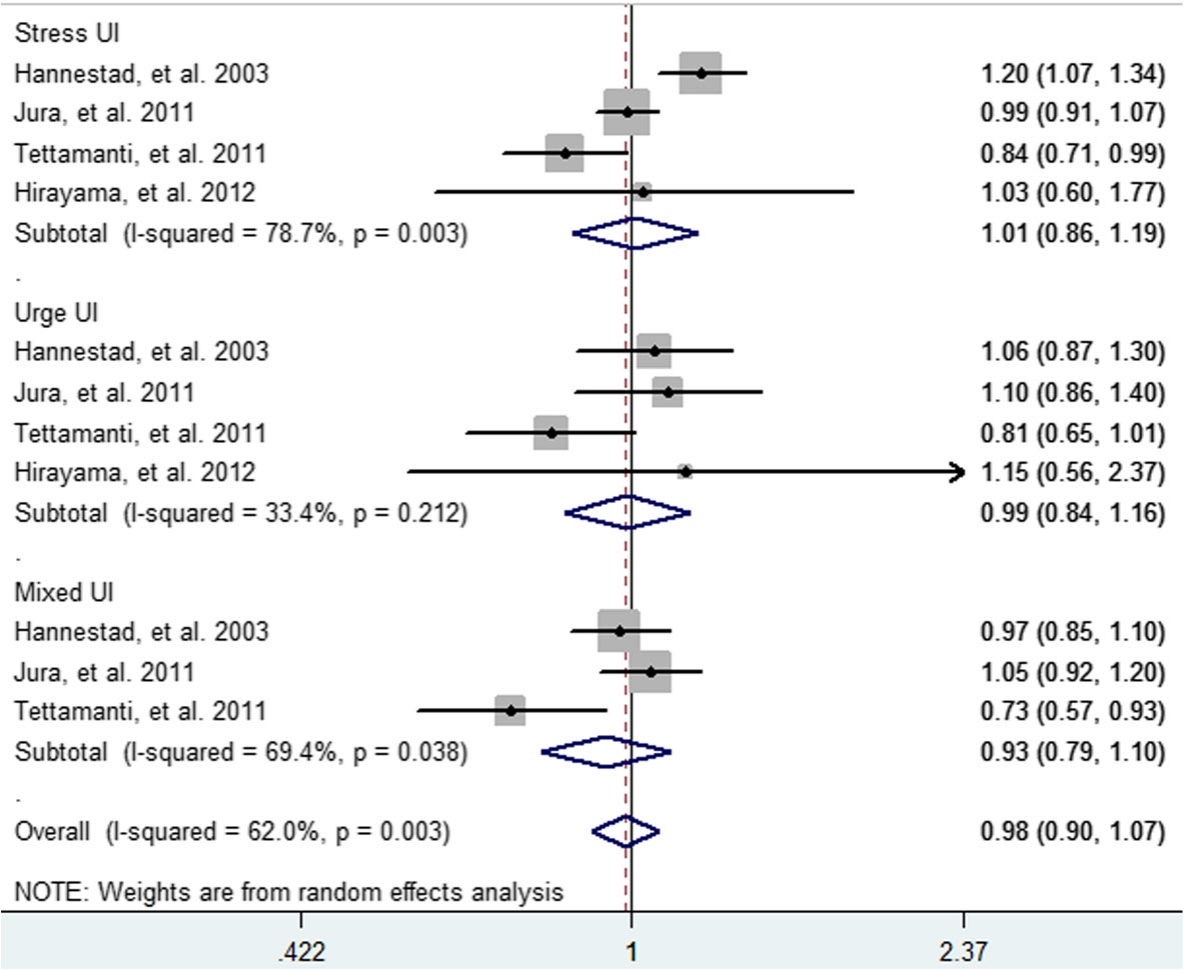
Forest plots of UI risk by subtype associated with coffee/caffeine consumption

For stress UI, the combined OR was 1.01 (95 % CI 0.86–1.19) (Fig. 6). For urge UI, the summary OR was 0.99 (95 % CI 0.84–1.16). For mixed UI, the pooled OR was 0.93 (95 % CI 0.79 to 1.10). For the different subtypes of incontinence, we did not observe significant association between coffee/caffeine intake and risk of UI.

### Sensitivity analysis

As for sensitivity analysis, we removed one study at a time and analyzed the rest. After excluding the study which carried the most weight [11], the OR was 1.01 (95 % CI 0.77–1.32). After excluding the study which carried the least weight [19], the OR was 0.98 (95 % CI 0.83–1.16).

### Publication bias

**Fig. 7:**
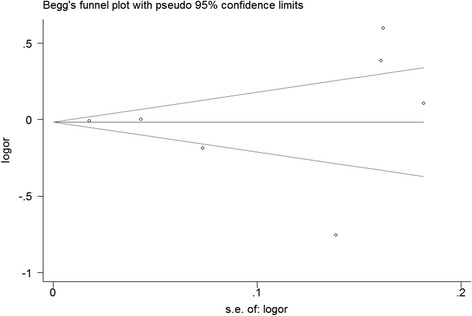
Funnel plot for studies of coffee/caffeine consumption in relation to UI risk

No funnel plot asymmetry was observed for the relationship between coffee/caffeine and UI. *P* values for Egger’s regression asymmetry test was 0.998 and the Begg’s adjusted rank correlation test was 0.764, indicating a low probability of publication bias (Fig. 7).

## Discussion

To our knowledge, this is the first meta-analysis to explore the association between coffee/caffeine intake and UI. We observed that coffee/caffeine consumption was not significantly associated with risk of overall UI. After deleting one study at a time and analyzing the rest, the summary OR ranged from 1.01 (95 % CI 0.77–1.32) to 0.98 (95 % CI 0.83–1.16). When evaluating the severity of UI symptoms, we found no relationship between coffee/caffeine consumption and moderate/severe UI. Moreover, coffee/caffeine consumption was not associated with types of UI (stress, urge, and mixed UI) when controlling for other UI risk factors.

Creighton and Stanton observed a statistically significant increase in detrusor pressure on bladder filling following administration of caffeine in women with detrusor instability [21]. Tomlinson et al. reported that the relationship between a decrease in the amount of dietary caffeine consumed and fewer daytime episodes of involuntary urine loss approached significance [22]. Thus, the relationship between lower urinary tract dysfunction and coffee/caffeine intake might be plausible. Considering that coffee/caffeine may exacerbate urinary incontinence, physicians often recommend a reduction in coffee/caffeine intake for individuals with incontinence symptoms.

To ascertain the impact of cumulative dose of coffee/caffeine intake on the risk of UI, we used a meta-analytic approach to estimate overall OR and 95 % CIs for regular coffee/caffeine drinkers versus individuals who seldom drank coffee/caffeine. In the seven studies, the lowest level of coffee/caffeine intake was defined as ‘never drink coffee’, whereas the highest level of coffee/caffeine intake was defined as ‘regularly drink coffee’. Of note, regular coffee/caffeine drinkers experienced an increased risk of 18 % for UI. However, no significant difference was found between the two groups.

According to the Incontinence Severity Index or other items, UI was categorized as “any” or “moderate/severe”. Three studies provided results on risk of moderate/severe UI [10, 11, 20], and one study reported the risk of frequent UI among women with daily caffeine intakes [12]. Jura stated that frequent incontinence was UI at least once per week among incident cases. Thus, frequent UI was also regarded as moderate/severe UI in the subgroup meta-analysis.

We also explored the association between coffee/caffeine consumption and incidence of UI by gender. Tettamanti and colleagues reported that women with a high coffee intake were at lower risk of any urinary incontinence compared with women not drinking coffee [13]. Gleason et al. found that caffeine intake ≥ 204 mg/day was associated with any UI in United States women [20]. A case-control study of Japanese adults failed to find an association between coffee/caffeine intake and incidence of UI [19]. Davis et al. demonstrated that caffeine consumption was significantly associated with moderate to severe urinary incontinence in United States men [12]. However, in stratified analyses by gender, no significant association was found between coffee/caffeine consumption and UI risk in both men and women.

We also analyzed type of incontinence as outcome. Four studies provided results on risk of UI specific for type (stress, urge, and mixed UI). To the best of our knowledge, the present study is the first meta-analysis that summarized the association between coffee consumption and risk of UI by type. Hannestad and colleagues stated that coffee intake was associated with an increased risk of stress UI [10]. However, we did not observe any significant (positive or negative) relations with UI subtypes in our study.

There are several strengths in the present meta-analysis. First of all, when different ORs were provided according to the different levels of coffee/caffeine consumption, we could combine the results of subgroups and calculated a common OR. Secondly, through visual inspection of a funnel plot and Begg’s and Egger’s tests, we observed no evidence of publication bias. Moreover, our findings were robust and reliable based on the consistent results from sensitivity analysis.

Some limitations in our study should be of concern. Firstly, adjusted confounding factors varied among different studies. Several potential confounding factors such as parity, BMI, smoking and water intake were not considered in several articles. Secondly, although no significant evidence of publication bias was observed, publication bias might be inevitable due to unpublished studies or original data. Thirdly, categories of coffee/caffeine intake varied from articles, which might lead to significant heterogeneity. Fourthly, due to the lack of relevant studies, crucial influences of coffee/caffeine consumption, including duration of coffee/caffeine intake and type of coffee/caffeine, had not been studied enough. Furthermore, a dose-response analysis could not be carried out due to the limited data provided by the included studies.

## Conclusion

In summary, to our knowledge, this is the first meta-analysis to date on the association between coffee/caffeine intake and risk of UI. The results from this meta-analysis of observational studies demonstrated that coffee/caffeine consumption was not associated with overall UI risk. Nevertheless, because of the potential limitations of this meta-analysis, conclusions must be drawn with caution, and more well-designed studies with large sample sizes should be conducted for further validation.
